# Knockdown of PR-DUB subunit *calypso* in the developing *Drosophila* eye and wing results in mis-patterned tissues with altered size and shape

**DOI:** 10.1093/g3journal/jkaf227

**Published:** 2025-09-29

**Authors:** Max Luf, Priya Begani, Anne M Bowcock, Cathie M Pfleger

**Affiliations:** Department of Oncological Sciences, Icahn School of Medicine at Mount Sinai, New York, NY 10029, United States; The Tisch Cancer Institute, Icahn School of Medicine at Mount Sinai, New York, NY 10029, United States; Department of Oncological Sciences, Icahn School of Medicine at Mount Sinai, New York, NY 10029, United States; The Tisch Cancer Institute, Icahn School of Medicine at Mount Sinai, New York, NY 10029, United States; The Graduate School of Biomedical Sciences, Icahn School of Medicine at Mount Sinai, New York, NY 10029, United States; Department of Oncological Sciences, Icahn School of Medicine at Mount Sinai, New York, NY 10029, United States; The Tisch Cancer Institute, Icahn School of Medicine at Mount Sinai, New York, NY 10029, United States; The Graduate School of Biomedical Sciences, Icahn School of Medicine at Mount Sinai, New York, NY 10029, United States; Department of Genetics & Genomics, Icahn School of Medicine at Mount Sinai, New York, NY 10029, United States; Department of Dermatology, Icahn School of Medicine at Mount Sinai, New York, NY 10029, United States; Department of Oncological Sciences, Icahn School of Medicine at Mount Sinai, New York, NY 10029, United States; The Tisch Cancer Institute, Icahn School of Medicine at Mount Sinai, New York, NY 10029, United States; The Graduate School of Biomedical Sciences, Icahn School of Medicine at Mount Sinai, New York, NY 10029, United States

**Keywords:** BAP1, calypso, polycomb repression, PR-DUB, *Drosophila*

## Abstract

The deubiquitinating enzyme BAP1 is the catalytic subunit of the Polycomb Repressive Deubiquitinase (PR-DUB) complex, which acts with the Polycomb Repressive Complexes 1 and 2 to regulate chromatin organization to repress homeotic genes and other developmental regulators. Loss of BAP1 is implicated in several cancers, in the familial cancer syndrome BAP1 Tumor Predisposition Syndrome, and in the neurodevelopmental disorder Küry-Isidor syndrome. In *Drosophila*, there are numerous reports in the literature describing developmental patterning phenotypes for several chromatin regulators, including the discovery of Polycomb itself, but corresponding adult morphological phenotypes due to developmental dysregulation of the *Drosophila BAP1* ortholog *calypso* (*caly*) are less well-described. We report here that knockdown of *caly* in the eye and wing produces concomitant chromatin dysregulation phenotypes. RNAi to *caly* in the early eye reduces survival and leads to changes in eye size and shape including eye outgrowths, some of which resemble homeotic transformations, whereas others resemble tumor-like outgrowths seen in other fly cancer models. Mosaic eyes containing *caly* loss-of-function tissue phenocopy *caly* RNAi. Knocking down *caly* across the wing disrupts wing shape and patterning, including effects on wing vein pattern. This phenotypic characterization reinforces the growing body of literature detailing developmental mis-patterning driven by chromatin dysregulation and serves as a baseline for future mechanistic studies to understand the role of BAP1 in development and disease.

## Introduction

**Fig. 1.**
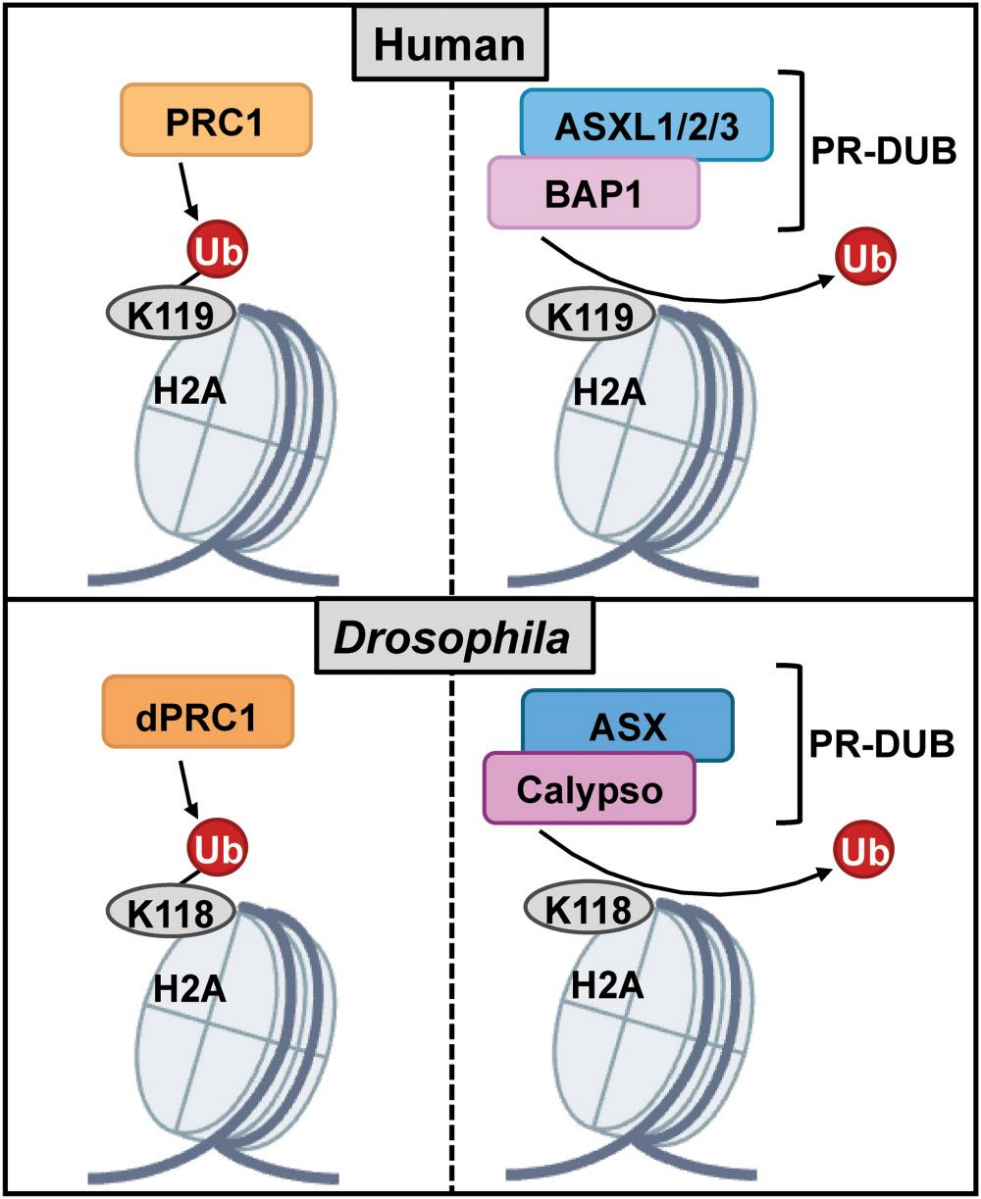
PR-DUB catalytic subunit *caly/BAP1* promotes H2A deubiquitination within the polycomb repressive system. Schematic representation of PRC1-mediated monoubiquitination of histone H2A and subsequent removal by the Polycomb Repressive Deubiquitinase (PR-DUB) complex in humans (top panels) and *Drosophila* (bottom panels). In humans, Polycomb Repressive Complex 1 (PRC1) catalyzes monoubiquitination of H2A at lysine 119 (H2AK119Ub), whereas in *Drosophila*, its functional ortholog dPRC1 targets lysine 118 (H2AK118Ub). These marks are removed by the PR-DUB complex, which comprises BAP1 and ASXL1/2/3 in humans, and Calypso and ASX in *Drosophila*. While the depicted complexes for PR-DUB represent the core catalytic units, both mammalian and *Drosophila* PR-DUB complexes associate with additional cofactors that modulate complex assembly, chromatin targeting, and enzymatic activity. PRC1 is made up of a variety of different components, which are depicted here as one complex.

The deubiquitinating enzyme (DUB) BAP1, BRCA1-associated protein 1, is a major tumor driver and metastasis suppressor in uveal melanoma (UM), the most common primary cancer of the eye (Kashyap et al. 2016; Jager et al. 2020). *BAP1* is mutated in 45% of UM and in 85% of UM that metastasize (Harbour et al. 2010; Robertson et al. 2017; Field et al. 2018). In addition, various other cancers have significant loss of *BAP1*, including mesothelioma (Bott et al. 2011; Testa et al. 2011; Wiesner et al. 2011), clear cell renal cancer (Ricketts et al. 2018), and cholangiocarcinoma (Jiao et al. 2013). Germline mutations in *BAP1* also lead to a *BAP1* Tumor Predisposition Syndrome (BAP1-TPS) (Bergman et al. 2006; Carbone et al. 2012, 2015). BAP1 is reported to regulate cell proliferation (Machida et al. 2009), cell death (Bononi et al. 2017), and nuclear processes crucial for genome stability, such as DNA repair and replication (Yu et al. 2014). The Polycomb repressive system is composed of 3 main protein complexes: Polycomb Repressive Complex 1 (PRC1), Polycomb Repressive Complex 2 (PRC2), and the Polycomb Repressive Deubiquitinase (PR-DUB) complex, in which BAP1 is the catalytic subunit (Scheuermann et al. 2010) and partners with additional sex combs-like (ASXL) protein (schematic in Fig. 1). These complexes repress homeotic (HOX) and other developmental regulator genes in cells where they must stay inactive, thereby ensuring proper differentiation and cellular identity throughout development (Scheuermann et al. 2010). Loss of essential components of these complexes can result in dysregulation of chromatin organization, which leads to developmental abnormalities and diseases such as cancer. Within the PR-DUB complex, BAP1 globally deubiquitinates lysine 119 on histone H2A (Fig. 1), and its loss leads to pervasive H2AK119ub1. Failure to constrain pervasive H2AK119ub1 titrates away Polycomb Repressive Complexes (PRC) from their targets, decreasing promoter H3K27me3 concentration and leading to chromatin compaction (Daou et al. 2015; Conway et al. 2021; Fursova et al. 2021). Despite these insights, the specific role of loss of BAP1 function in the development of different cancers and their metastases is unclear.

Model systems such as *Drosophila* provide a useful context to further study BAP1 function and its roles in vivo. In fact, *polycomb* itself was originally discovered in *Drosophila* (Lewis and Mislove 1947). PR-DUB subunits *BAP1* and *ASXL* are highly conserved and represented in *Drosophila* by *calypso* (*caly*, also called *dBap1*) and *Additional sex combs* (*Asx*), respectively. Extensive literature in *Drosophila* describes the role of PRC1, PRC2, a number of Polycomb regulators, and the other PR-DUB subunit *Asx*, but less work has been done to characterize *caly* developmental phenotypes. Previous work in flies led to the isolation of *caly* mutant alleles that were based on the ability of mutant clones in the wing to phenocopy Polycomb Group (PcG) transformations (de Ayala Alonso et al. 2007). This showed that loss of *caly* catalytic activity increases monoubiquitinated H2A in vitro and decreases repression of PcG target genes and *HOX* genes in vivo in clones in the wing, as seen in human cell lines and murine models (Scheuermann et al. 2010). Substantial characterization of embryos and larval structures, including immunostaining analyses that revealed detailed patterns of dysregulating signaling components and homeotic regulators in larval clones homozygous for either *Asx* or *caly* mutant alleles, has revealed substantial mechanistic insights (Halachmi et al. 2007; Bischoff et al. 2009; Bonnet et al. 2022; Brown et al. 2023, 2025). In addition to these extensive characterizations of developmental structures, some eye and other epidermal morphological phenotypes in adults that resulted from these random mutant clones or *caly* RNAi have been mentioned in previous reports (Halachmi et al. 2007; Bonnet et al. 2022; Brown et al. 2023, 2025). However, the literature is generally lacking in detailed morphological descriptions of adult structures resulting from tissue-wide loss of *caly* in development that would be important for future work to model BAP1-associated health concerns, and specifically lacking such detailed reports for RNAi allele *caly^HMC04109^* across a range of gal4 drivers.

Given the importance of *BAP1* in suppression of tumorigenesis and metastasis, it is important to complement the characterizations of other chromatin regulators and clonal analysis of *caly* in development reported in published literature with a detailed description of how *caly* knockdown across different tissue-wide contexts in development affects patterning and gross morphology. This will enable us to further understand the role of *caly* in development and prioritize contexts for further study to elucidate the role of *BAP1* as a tumor suppressor in human cancer.

Here, we characterize phenotypes arising from knockdown of *caly* using inducible RNAi allele *caly^HMC04109^* in developing tissues. RNAi to *caly* in multiple contexts in the developing eye and wing increased lethality and led to a spectrum of phenotypes in surviving flies. RNAi in the early eye resulted in a range of reduced eye sizes and outgrowths, some of which differentiated into structures that resembled antennae, maxillary palps, or even legs. Other outgrowths were difficult to classify and resembled tumor-like outgrowths seen in other fly cancer models. In contrast, RNAi in the differentiating eye results in eyes of slightly increased size that appeared normal morphologically. RNAi in the wing led to abnormalities affecting patterning, including wing vein phenotypes, blisters, and crumpling. These phenotypes are similar to those reported due to loss of function of other chromatin regulators and validate the allele *caly^HMC04109^* as a tool in future mechanistic studies to understand the role of BAP1 in disease and cancer.

## Materials and methods

### Rigor and reproducibility

The reported work represents reproducible experiments that reflect a minimum of 3 well-controlled, independent trials. For most experiments, at least one set of trials was done by a different lab member than the other 2 sets of trials to reduce the chance of replicating unintended observer bias.

### Statistical analysis

**Fig. 2.**
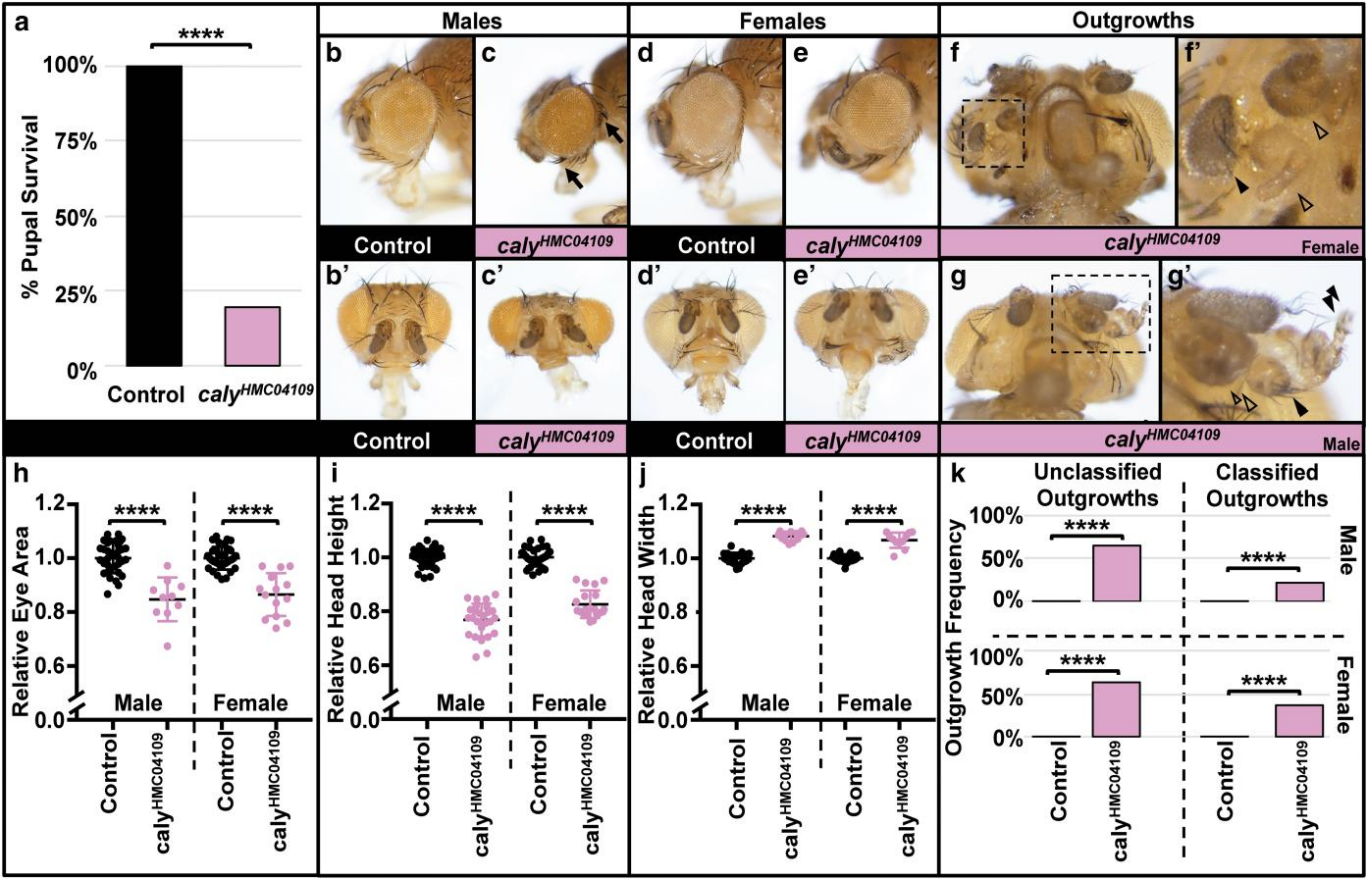
RNAi to *caly* using *ey-gal4* reduces pupal survival, causes eye shape and size phenotypes, and causes tissue outgrowths. Experiments were performed at 25 °C. a) Graph summarizing the percent pupal survival for control *ey-gal4/+* flies (left) vs driving RNAi to *caly* using *ey-gal4* and *caly^HMC04109^* (right). RNAi to *caly* dramatically reduces survival of pupae to adulthood. **** indicates *P* < 0.0001 in both chi-square and Fisher's exact tests. b to g´) eyes/heads in b to c´ and g to g´ show males, and images in d to f´ show females. Eyes in b, c, d, and e are at the same scale to allow for comparisons to each other; eyes in b´, c´, d´, and e´ are at the same scale to allow for comparisons to each other. b, d) *ey-gal4/+* eye profile showing control eye shape and size. b´, d´) Anterior images of heads of the genotypes shown in b and d. c, e) RNAi to *caly* using *ey-gal4* (ey > *caly^HMC04109^*) causes a reduction in eye size, rounder eye shape, and change in bristle pattern (highlighted by the solid arrows in c). c´, e´) Anterior view highlights that the head is shorter but wider. f to g´) In addition to eye size and shape changes, eyes contain a variety of different outgrowths, including outgrowths that appear to have differentiated into structures resembling antennae, maxillary palps, or legs (solid arrowheads), whereas others seem to lack obvious morphology associated with specific structures and cannot be classified morphologically based on visual inspection (arrowhead without fill). Additional view of outgrowths from the head in f to f´ is shown in Supplementary Fig. 1e–1e´, and additional images of ey > *caly^HMC04109^* heads are shown in Supplementary Fig. 1, including an outgrowth of almost an entire leg from the eye in Supplementary Fig. 1c. h to j) Graphs summarizing h) relative eye area, i) relative head height, and j) relative head width for *ey-gal4/+* control males (first lane) and females (third lane) vs ey > *caly^HMC04109^* males (second lane) and females (fourth lane). **** indicates *P* < 0.0001 in *t* tests. Eye image indicating how head height and width were measured is shown in Supplementary Fig. 2a. To highlight the head shape changes, we also graphed the ratio of width-to-height in Supplementary Fig. 2b. k) Graphs quantifying the percent of eyes with outgrowths in *ey-gal4/+* controls (left lanes) vs ey > *caly^HMC04109^* (right lanes) when the outgrowths have unclassifiable outgrowths (left graphs) or outgrowths with classifiable morphology (such as legs, antennae, or palps) (right graphs) for males (upper graphs) and females (bottom graphs). **** indicates *P* < 0.0001 in both chi-square and Fisher's exact tests.

**Fig. 3.**
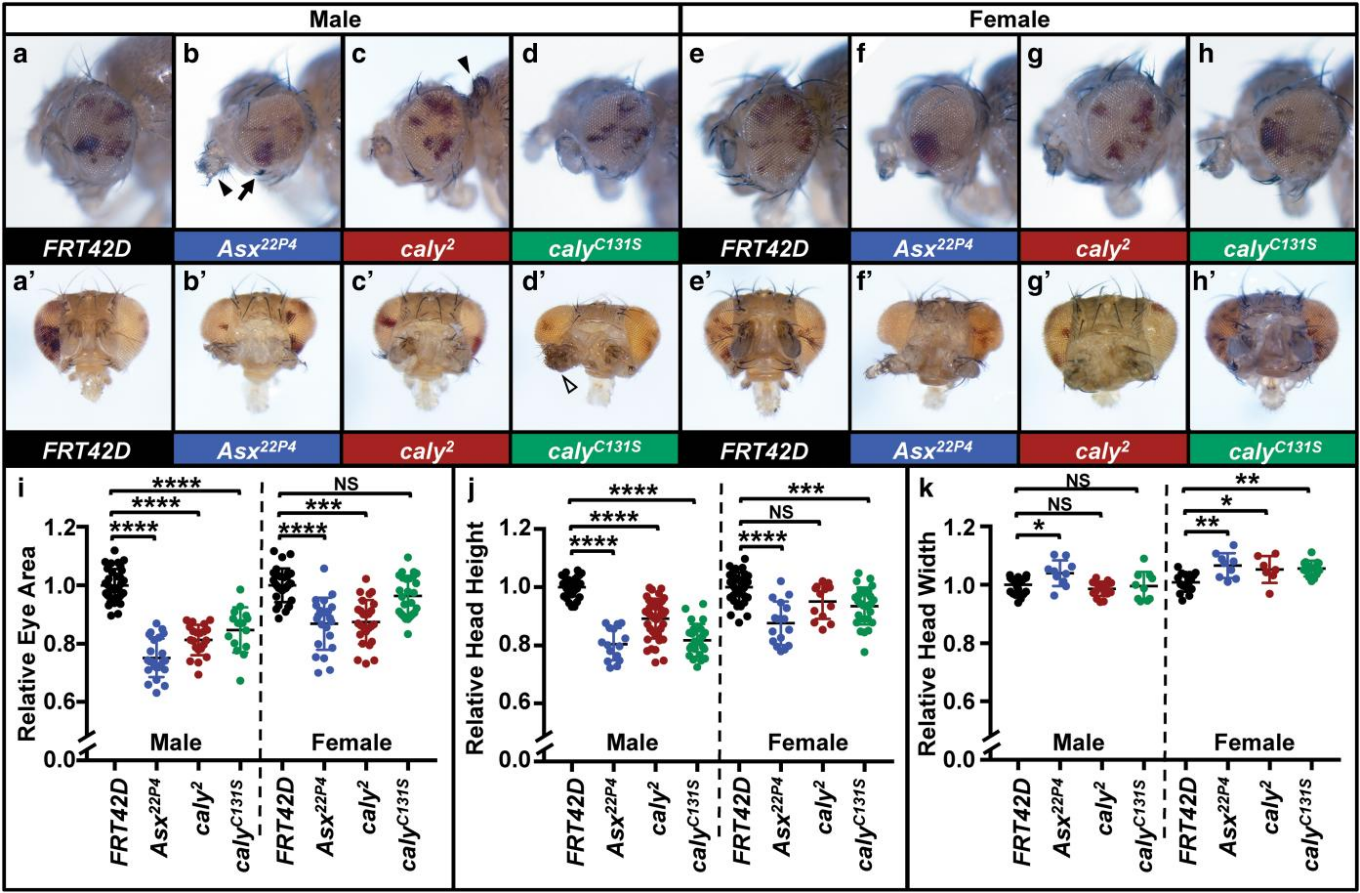
Eyes composed largely of homozygous mutant *caly* tissue in the eye phenocopies *caly* RNAi by changing eye shape and size, and causing eye outgrowths. Experiments were performed at 25 °C. Images in a to d´ show males and in e to h´ show females generated using *eyFLP*, an *FRT42D* chromosome with cell-lethal mutation l(2)R11^1^ and a pW+ insertion, and an *FRT42D* chromosome either wild-type or containing mutations in *Asx* or *caly*. Due to the cell-lethal mutation, tissue homozygous for the l(2)R11^1^ chromosome dies. Remaining tissue is either red heterozygous tissue that did not undergo mitotic recombination or white homozygous wildtype, *Asx*, or *caly* mutant tissue. Due to the lighting, white tissue in these images appears to have a yellow or orange hue. Eyes in a, b, c, d, e, f, g, and h are at the same scale to allow for comparisons to each other; eyes in a´, b´, c´, d´, e´, f´, g´, and h´ are at the same scale to allow for comparisons to each other. a, e) Eyes containing *FRT42D* control tissue. a´, e´) Anterior view showing heads of the genotypes from a, e. b, f) Eyes containing *Asx^22P4^* mutant tissue. b´, f´) Anterior view showing heads of the genotypes from b, f. c, g) Eyes containing *caly^2^* null mutant tissue. (c´, g´) Anterior view showing heads of the genotypes from c, g. d, h) Eyes containing *caly^C131S^* catalytically inactive mutant tissue. d´, h´) Anterior view showing heads of the genotypes from d, h. Eyes containing *Asx* and *caly* mutant tissue are round and smaller with bristle abnormalities (example with an arrow in b) and with occasional outgrowths that differentiate into structures resembling antennal or other morphology (example with a solid arrowhead in b) or with no clear morphology (example with an open arrowhead in d´). i to k) Graphs summarizing i) relative eye area, j) relative head height, and k) relative head width for eyes containing control tissue (lanes 1 and 5, black), *Asx^22P4^* mutant tissue (lanes 2 and 6), *caly^2^* mutant tissue (lanes 3 and 7), and *caly^C131S^* mutant tissue (lanes 4 and 8). Eye image indicating how head height and width were measured is shown in Supplementary Fig. 2a. Males are shown in lanes 1 to 4 and females in lanes 5 to 8 for each graph. As with *caly* RNAi in Fig. 1, heads with *Asx* or *caly* mutant tissue have smaller eyes, reduced height, and increased width compared to controls. To highlight the head shape changes, we graphed the ratio of width-to-height in Supplementary Fig. 2c. In i to k, NS indicates not significant, * indicates *P* < 0.05, ** indicates *P* < 0.01, *** indicates *P* < 0.001, and **** indicates *P* < 0.0001.

**Fig. 4.**
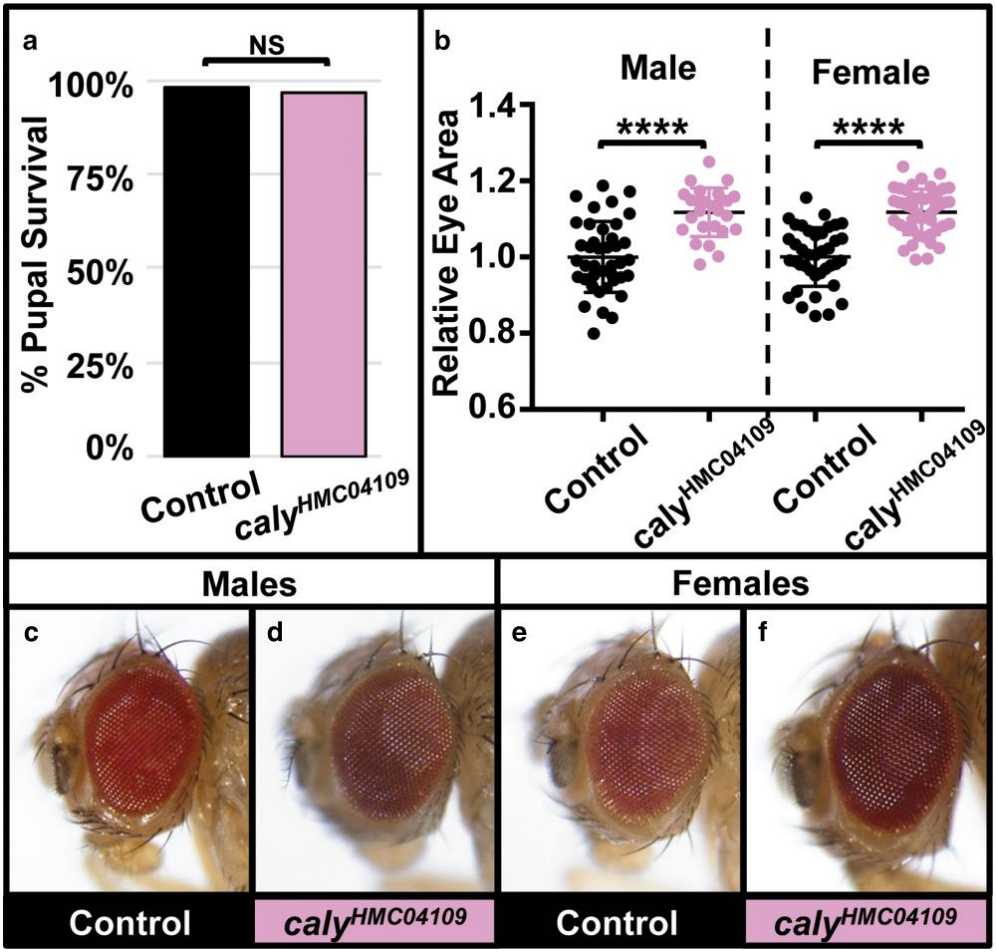
RNAi to *caly* using *GMR-gal4* does not affect morphology but causes increased eye size. Experiments in this figure were performed at 25 °C. a) Graph summarizing the percent pupal survival in control *GMR-gal4/+* flies (left) vs driving RNAi to *caly* using *GMR-gal4* and *caly^HMC04109^* (*GMR > caly^HMC04109^*) (right). NS indicates not significant in both chi-square (*P* = 0.087) and Fisher's (*P* = 0.12) exact tests. b) Graph showing relative eye area in males (left 2 lanes) and females (right 2 lanes) of control GMR-gal4/+ flies (first and third lanes) and *GMR > caly^HMC04109^* flies (second and fourth lanes) relative eye area males and females. Eye size increases by approximately 11.2%. The eye morphology appears normal despite the increased size. **** indicates *P* < 0.0001 in *t* tests. c, e) Control *GMR-gal4/+* eyes. d, f) *GMR > caly^HMC04109^* eye. Eyes in c, d, e, and f are at the same scale to allow for comparisons to each other.

**Fig. 5.**
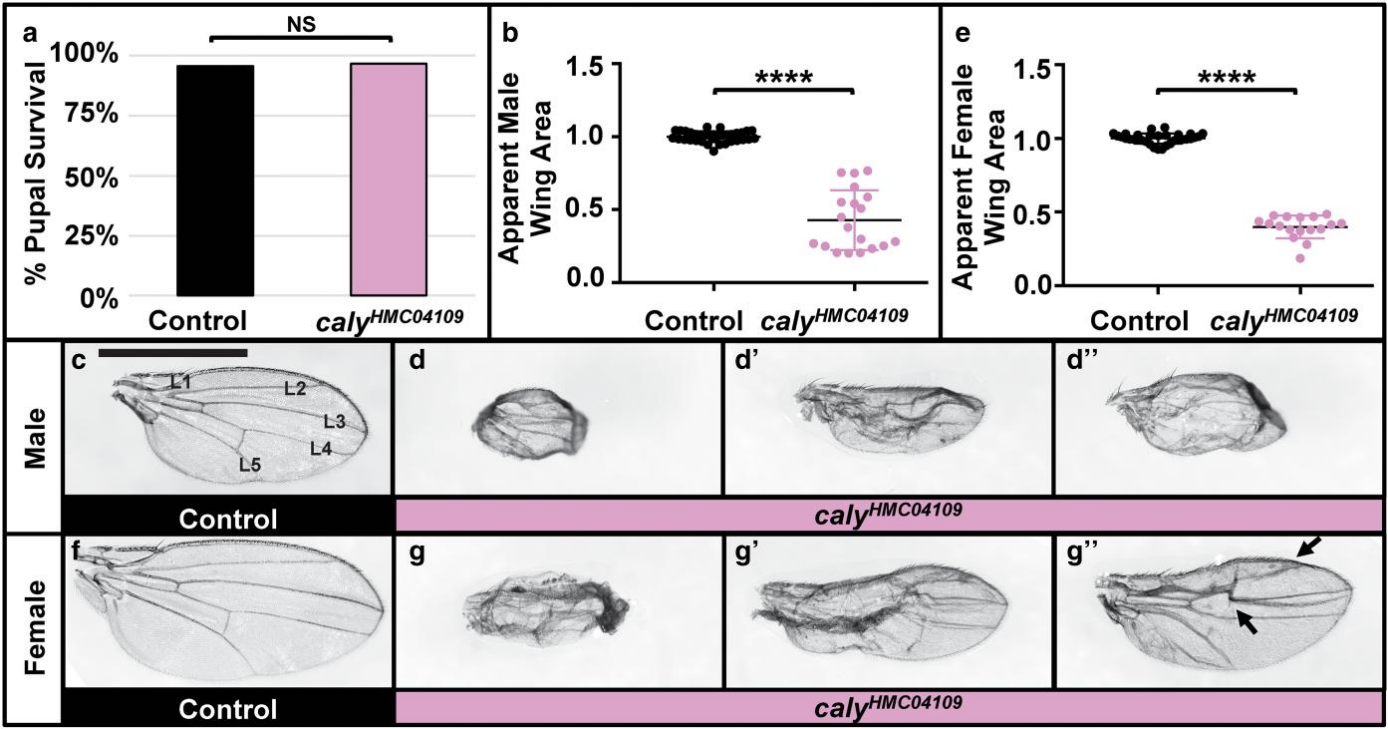
RNAi to *caly* using *ms1096-gal4* disrupts wing patterning and reduces wing size. Experiments in this figure were performed at 25 °C. a) Graph summarizing the percent pupal survival in control *ms1096-gal4/+* flies vs driving RNAi to *caly* using *ms1096-gal4* and *caly^HMC04109^* (*ms1096 > caly^HMC04109^*). RNAi to *caly* does not affect pupal survival. NS indicates not significant in chi-square (*P* = 0.7468) and Fisher's exact (*P* > 0.9999) tests. b to d´´) data for males; e to g´´) data for females. b, e) Relative apparent wing area decreases upon RNAi to *caly* (right) compared to controls (left) for males (b) and females (e). c, f) Control *ms1096-gal4* wing. Wings in c to g´´ are at the same scale to allow for comparisons; scale bar in C represents 1 mm and applies to wings in c to d´´ and f to g´´. Wing veins L1 to L5 are labeled in C for clarity. d to d´´, g to g´´) Both male (d to d´´) and female (g to g´´) *ms1096 > caly^HMC04109^* wings have a range of phenotypes from very small wings with abnormal shapes including “cupping” of the wing or curling of the wing margins (d, g) to wings with moderate phenotypes that show less of a decrease in apparent wing area but still do not flatten properly even once inflated (d´, g´) to wings that with even milder phenotypes that still show abnormal patterning (arrows) (d´´, g´´).

**Fig. 6.**
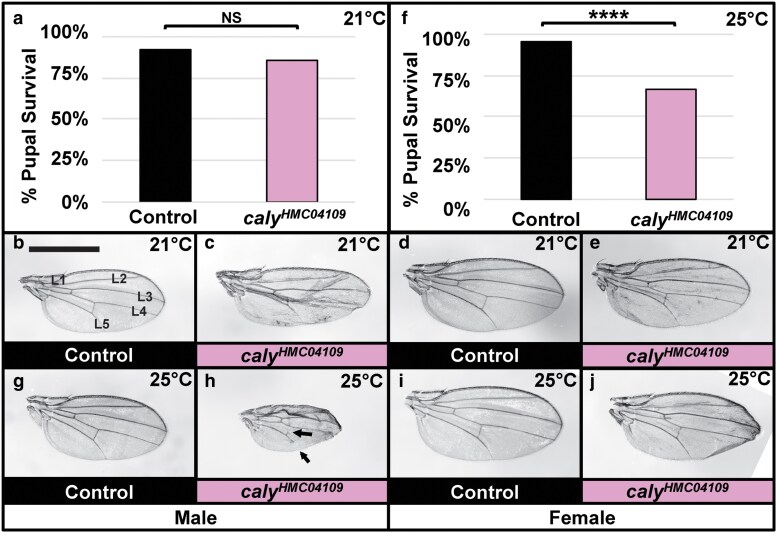
RNAi to *caly* across the wing using *c765-gal4* reduces survival and causes wing abnormalities. a to e) Experiments in a to e were performed at 21 °C. a) Graph summarizing the percent pupal survival in control *c765-gal4/+* flies vs driving RNAi to *caly* using *c765-gal4* and *caly^HMC04109^* (*c765 > caly^HMC04109^*). There is a reproducible trend of decreased survival upon *caly* RNAi, but this is not statistically significant. NS indicates not significant in chi-square (*P* = 0.1788) and Fisher's exact (*P* = 0.2577) tests. b, d) Control *c765-gal4/+* wing. Wings in b to e and g to j are at the same scale to allow for comparisons; scale bar in b represents 1 mm and applies to wings in b to e and g to j. Wing veins L1 to L5 are labeled in b for clarity, c, e) c765 > *caly^HMC04109^* wing. c765 > *caly^HMC04109^* wings have disrupted wing patterning. Male wings are shown in b and c, and female wings in d and e. f to j) Experiments in f to j were performed at 25 °C. f) Graph summarizing the percent pupal survival in control *c765-gal4/+* flies vs c765 > *caly^HMC04109^* flies. Reproducibly, there is statistically significant decreased survival upon *caly* RNAi at the higher temperature of 25 °C compared to the survival at 21 °C shown in a. **** indicates *P* < 0.0001 in both chi-square and Fisher's exact tests. g, i) *c765-gal4/+* wing. h, j) c765 > *caly^HMC04109^* wing. RNAi to *caly* disrupts wing patterning including occasional loss of longitudinal or crossvein material (arrows) and causes curling at the wing margin; this is more common in male wings. Male wings are shown in g and h, and female wings in i and j.

Eye area, head height, head width, and wing area were measured with ImageJ software. Raw area measurements in pixels were normalized in order to facilitate comparisons between genotypes (as is the standard in the field) using Excel by calculating the mean of the data from the control genotype and then dividing individual data points for the control genotype and other genotypes by the mean of the control genotype in Excel. Raw survival data consisting of flies surviving to adulthood vs those that died before adulthood were normalized by dividing the number in each category by the total number of flies (both surviving and not surviving) and multiplying by 100 to establish percentages. Normalized measurements and survival data were then graphed using Excel (Figs. 2a, k, 4a, 5a, and 6a, f) and GraphPad Prism (Figs. 2h to j, 3i to k, 4b, and 5b, e). Categorical analysis to analyze survival (Figs. 2a, 4a, 5a, and 6a, F) or type of outgrowths (Fig. 2k) used chi-square and Fisher’s exact tests as appropriate, calculated in GraphPad Prism. *t* test (Figs. 2h to j, 4b, and 5d, e) and one-way ANOVA analysis with multiple comparisons (Fig. 3i to k) assessed changes in eye area, head height, head width, or wing size. *P*-values, raw measurements, and normalized values are listed in Supplementary File 1.

### *Drosophila* experiments

Crosses at the indicated temperatures were set up on standard *Drosophila* medium as in our previous work (Yan et al. 2009; Yan et al. 2010; Washington et al. 2020; Reimels et al. 2024). In each trial for each experiment, crosses used food prepared in the same batch and were incubated in close proximity to experience the same environment to rule out unintended environmental variables or food batch variations. Gal4 drivers were obtained from the Bloomington *Drosophila* Stock center or other labs in the *Drosophila* community (for details, please see Table 1). *caly* RNAi caly^HMC04109^, and cell lethal *l(2)cl-R11^1^* were from the Bloomington Stock center, and *caly* and *Asx* alleles were graciously provided by Dr. J. Müller (de Ayala Alonso et al. 2007; Bonnet et al. 2022).

**Table 1.** Table of reagents used with corresponding identifiers.

Drosophila strains		
Strain	Source	Identifier
*w^1118^*	The fly community and Bloomington *Drosophila* Stock Center (BDSC)	BL-3605, BL-5905 and others RRID:BDSC_3605, RRID:BDSC_5905
*caly* ^HMC04109^	BDSC	Can be obtained from BDSC, 56888 RRID:BDSC_56888
*ey-gal4*	Hariharan lab	
*c765-gal4*	BDSC, NYC fly community	Can be obtained from BDSC, BL-36523 RRID:BDSC_36523
*ms1096-gal4*	BDSC	Can be obtained from BDSC, BL-8696 RRID:BDSC_8696
*GMR-gal4*	BDSC	Can be obtained from BDSC, BL-8605 RRID:BDSC_8605
*FRT42D*	BDSC	Can be obtained from BDSC, BL-5626 RRID:BDSC_5626
*y, w, eyFLP, GMR-lacZ; FRT42D, l(2)cl-R11^1^, w+/CyO*	BDSC	Can be obtained from BDSC, BL-5617 RRID:BDSC_5617
*FRT40, FRT42D y+caly^2^/CyO,ubi-GFP*	Gift from Müller Lab	
*FRT40, FRT42D y+caly^C131S^/CyO,ubi-GFP*	Gift from Müller Lab	
*FRT40, FRT42D y*+ *Asx^22P4^/CyO,ubi-GFP*	Gift from Müller Lab	

Software	
Program	Source
Image J	https://imagej.net/ij/
GraphPad Prism	https://www.graphpad.com/scientific-software/prism/
Microsoft Excel	https://www.microsoft.com/en-us/microsoft-365/excel

### Pupal lethality/survival experiments

For survival experiments, pupal cases of the indicated genotypes were scored as dead (in which dead pupae remained in the pupal cases) or empty (from which surviving flies had eclosed) and counted at 26 d (21 °C) or 18 d (25 °C) as in our previous study (Singh et al. 2023). Dead pupal cases are easily distinguished from empty pupal cases or from developing pupae that are still alive and have not yet eclosed.

### Image analysis and processing

Adult eyes and wings were photographed using a Nikon DS-Fi3 microscope camera and saved as TIFF files. All eyes and wings within each trial of an individual experiment were photographed at the same magnification to allow for images to be compared to each other to determine relative size. Raw wing images were converted to grayscale and cropped in Adobe Photoshop. The same degree of resizing and cropping were applied in parallel to all images from the same experiment to maintain the ability for figure panels to be compared. A scale bar equivalent to 1 mm is applied to the first control wing in Figs. 5 and 6 and applies to all the wings in the corresponding set. Brightness and contrast of eye and wing images were adjusted in Adobe Photoshop to maximize clarity; adjustments were applied to the entire images. Genotypes are summarized below and identifiers are listed in Table 1.

### Genotypes of flies in images or graphs

*w; ey-gal4/+* (Fig. 2b to b´, d to d´; left genotype in graphs in Fig. 2a, h to k, Supplementary Fig. 2b)
*w; ey-gal4/caly^HMC04019^* (Fig. 2c to c´, e to e´, f to g´, Supplementary Fig. 1a to e´; right genotype in graphs in Fig. 2a, h to k, Supplementary Fig. 2b)
*y w eyFLP; FRT42D/FRT42D* l(2)cl-R11^1^ (Fig. 3a to a´, e to e´; left-most genotype in graphs in 3i to k, Supplementary Fig. 2c)
*y w eyFLP; FRT42D Asx^22P4^/FRT42D* l(2)cl-R11^1^ (Fig. 3b to b´, f to f´; second genotype in graphs in 3i to k, Supplementary Fig. 2c)
*y w eyFLP; FRT42D caly^2^/FRT42D* l(2)cl-R11^1^ (Fig. 3c to c´, g to g´; third genotype in graphs in 3i to k, Supplementary Fig. 2c)
*y w eyFLP; FRT42D caly^C131S^/FRT42D* l(2)cl-R11^1^ (Fig. 3d to d´, h to h´; right-most genotype in graphs in 3i to k, Supplementary Fig. 2c)
*w; GMR-gal4/+* (Fig. 4c and e; left genotype in graphs in Fig. 4a and b)
*w; GMR-gal4/caly^HMC04019^* (Fig. 4d and f; right genotype in graphs in Fig. 4a and b)
*w, ms1096-gal4* (Fig. 5c, left genotype in graph in Fig. 5a and b)
*w, ms1096-gal4/+* (Fig. 5f, left genotype in graph in Fig. 5a and e)
*ms0196-gal4; caly^HMC04019^/+* (Fig. 5d to d´´; right genotype in graph in 5a and b)
*ms0196-gal4/+; caly^HMC04019^/+* (Fig. 5g to g´´; right genotype in graph in 5a and e)
*w; c765-gal4/+* (Fig. 6b, d, g, and i; left bar in graphs in Fig. 6a and f)
*w; caly^HMC04019^/+; c765-gal4/+* (Fig. 6c, e, h, and j; right bar in graphs in 6a and f)

## Results and discussion

Given the importance of BAP1 and the range of phenotypes reported for other Polycomb regulators in *Drosophila*, we utilized several Gal4 drivers that drive expression in the developing eye or wing to establish the phenotypes of RNAi allele *caly^HMC04109^*. Characterizing tissue-wide knockdown has the potential to (i) reinforce previous studies (e.g. Bonnet et al. 2022) that focused on characterizing *caly* phenotypes in development and mention the phenotypes in adult morphology due to random *caly* mutant clones and also (ii) reveal previously unreported phenotypes that require larger swaths of tissue undergoing *caly* knockdown to be observed and/or for which consistent knockdown in only one tissue rather than random clones is required for quantification.

### RNAi to or mutation in *caly* in the early eye caused lethality and a range of phenotypes

The driver *ey-gal4* has been reported to express in the early cells of the imaginal eye disc (Quiring et al. 1994; Halder et al. 1998; Hazelett et al. 1998) and also in other tissues, including the nervous system, larval brain, and genital discs (Adachi et al. 2003; Weasner et al. 2009). Inducing RNAi with *caly^HMC04109^* and *ey-gal4* resulted in substantial pupal lethality compared to controls (quantified in Fig. 2a). *ey > caly^HMC04109^* flies that survived to adulthood demonstrated a range of phenotypes (Fig. 2b to k) including eyes that appeared morphologically almost like control eyes as noted in a previous report (Brown et al. 2023) and rough eyes of reduced size (Fig. 2c to c´ for males, 2e to e´ for females; quantified in Fig. 2h; additional examples in Supplementary Fig. 1) compared to control eyes (Fig. 2b to b´ for males, Fig. 2d to d´ for females). The heads were reduced in height compared to controls (for anterior view of the eyes, Fig. 2c´ compared to 2b´ for males, Fig. 2e´ compared to 2d´ for females; quantified in Fig. 2i). In many cases, the eye tissue appeared to be bulging (Fig. 2c´ and e´), and despite the reduced area at the base of the eye (Fig. 2h), overall width of the head increased compared to controls (Fig. 2j). This is highlighted by an increase in the ratio of width to height compared to control eyes (Supplementary Fig. 2b). Most eyes showed bristle phenotypes in the anterior region in the periphery of the eye (arrows in Fig. 2c), and we also saw what appeared to be antennal duplications or other outgrowths (Fig. 2f to g´, Supplementary Fig. 1). Some of these outgrowths resembled specific differentiated structures like antennae, maxillary palps, or even legs due to morphological characteristics such as shape, bristle pattern, or even segmentation which we refer to as “classified outgrowths.” On one occasion, we saw almost an entire leg growing from the head with what may have been ommatidia on its distal tip (Supplementary Fig. 1c). Many of the “classified” outgrowths did not form complete structures, so the identity of the type of tissue forming was not always conclusive or unambiguous. Therefore, we did not further quantify these outgrowths based on presumed tissue identity. Curiously, the Brown et al. (2023) examination of imaginal eye discs driving *caly* RNAi using the same transgene reported ectopic expression of *Antp* posterior to the morphogenetic furrow that could explain some of these phenotypes. It is unclear why we see this range of phenotypes in adults that was not reported in Brown et al. (2023), despite the clear *Antp* dysregulation they saw for this genotype, which also reinforced similar Antp staining in *caly* mutant clones in mosaic discs previously reported (Bonnet et al., 2022) and which has also been seen for *caly* RNAi driven by *DE-gal4* (Brown et al., 2025). We speculate that the substantial lethality of this genotype could have resulted in lethality of such flies in that study under slightly different conditions. Similar outgrowths, including transformations into legs, were seen upon creating random clones of *caly* mutant tissue in antennae (Bonnet et al. 2022). Extensive immunostaining of such clones in larval discs confirmed HOX gene upregulation and homeotic transformation (Bonnet et al., 2022). In contrast to these homeotic outgrowths produced by random clones in that study, our *ey-gal4* mediated *caly* RNAi revealed additional overgrowths that lacked obvious morphological characteristics and thus were difficult to classify appearing as outgrown tissue covered by cuticle without other obviously identifiable features which we refer to as “unclassified outgrowths” (Fig. 2f to g´, Supplementary Fig. 1; quantification of relative “classified” vs “unclassified” outgrowths, Fig. 2k). We also observed outgrowths from tissue other than the antennae (Fig. 2f to f´). Previous reports of adult phenotypes resulting from random heat shock-induced clones throughout the eye and head described normal morphology for clones in regions of the head other than the antennae (Bonnet et al. 2022). Taken together with the variety of other phenotypes we observed from tissue-wide knockdown, including bristle abnormalities in the anterior region of the eye, eye area reduction, head shape phenotypes, and outgrowths outside the antennae, we speculate that some phenotypes require a minimum amount of *caly-*knocked down tissue. Alternate models include the possibility that wild-type tissue (absent in contexts of tissue-wide knockdown) may nonautonomously influence mutant tissue to correct patterning phenotypes or that the timing of the heat shock to induce random clones could differ from *ey-gal4*-mediated expression in ways necessary to elicit these phenotypes.

To establish if *ey > caly^HMC04109^* phenotypes resulted from knockdown of *caly* specifically and to rule out off-target effects of using this RNAi allele, we generated eyes containing primarily *caly* mutant tissue for null allele *caly^2^* (de Ayala Alonso et al. 2007) and for catalytically inactive allele *caly^C131S^* (Bonnet et al. 2022) by utilizing the FLP/FRT system and a cell-lethal mutation on the control 2R chromosome. *yweyFLP; FRT42D l(2)/40AFRT,FRT42D caly^2^* and *yweyFLP; FRT42D l(2)/40AFRT,FRT42D caly^C131S^* eyes contained heterozygous tissue (red, which did not undergo mitotic recombination) and primarily *caly* mutant tissue (white) (Fig. 3c to d´, g to h´). As in *ey > caly^HMC04109^* eyes (Fig. 2, Supplementary Figs. 1 and 2), these eyes were rough, round, and generally showed reduced eye area, reduced head height, and increased head width phenotypes (Fig. 3i to k) compared to control eyes (Fig. 3a to a´, e to e´, i to k) although this was not always statistically significant for the *caly* alleles as indicated in the figure. The lack of statistical significance could be due to the mosaic nature of the eyes, which also contained heterozygous tissue, rather than eye-wide *caly* knockdown produced when using *ey-gal4* or due to different degrees of knockdown of *caly* function. To highlight the head shape changes, we graphed the ratio of width-to-height in Supplementary Fig. 2c. These phenotypes also resembled eyes composed of primarily *Asx* mutant tissue, which we included as an additional control because Asx is the other subunit of PR-DUB (Fig. 3b to b´, f to f´). We also observed eye outgrowths (Fig. 3b and c). Eyes containing *Asx* and *caly* mutant tissue also exhibited bristle abnormalities and occasional outgrowths that differentiated into structures resembling antennae, legs, or other morphology (example in Fig. 3b) or with no clear morphology (example in Fig. 3d´). These outgrowths resembled those seen upon *caly* RNAi (Fig. 2 and Supplementary Fig. 1) and those seen previously upon generating random heat shock-induced homozygous clones of *caly^C131S^* (Bonnet et al. 2022) or homozygous clones of *Asx^ku256^* (Halachmi et al. 2007) in the antennae, but also sometimes occurred in regions outside the antennae (e.g. Fig. 3b), unlike those reported in these previous studies.

We cannot rule out that off-target effects contributed to the phenotypes in *ey > caly^HMC04109^* flies, but the similarity of *ey > caly^HMC04109^* phenotypes compared to eyes containing primarily *caly^2^* or *caly^C131S^* mutant tissue would be consistent with these phenotypes resulting from loss-of-function in *caly*, not due to RNAi off-target effects on other genes. The similarity in phenotypes compared to eyes containing primarily *Asx* mutant tissue would be consistent with these phenotypes resulting more specifically from loss of PR-DUB activity. These phenotypes also resembled the phenotypes of dysregulating chromatin and interfering with Polycomb group proteins, their targets, and Pax6 (Plaza et al. 2001; Nègre et al. 2006; Luque and Milán 2007; Arancio et al. 2010; Zhu et al. 2018), over-expression of OSA (Baig et al. 2010) or dysregulating other regulators of chromatin architecture like *Defective proventriculus* (*Dve*) (Puli et al. 2024). It will be important for future work to assess the levels and patterns of Pax6, OSA, Dve, and other Polycomb group targets in developing eye discs to establish if their dysregulation underlies these phenotypes. In fact, dysregulation of many chromatin regulators and loss of mis-expression of Polycomb targets also demonstrate similar outgrowths, some of which appear to take on differentiated morphology resembling other structures such as cuticle or leg outgrowths we saw here (Plaza et al. 2001; Dong et al. 2002; Dey et al. 2009; Puli et al. 2024) including the ectopic legs seen upon overexpressing of *Antennapedia* (*Antp*) (Schneuwly et al. 1987). This is also consistent with the ectopic *Antp* expression seen broadly posterior to the morphogenetic furrow and in the anterior region of the antennal eye-antennal discs upon loss of *caly or Asx* (Halachmi et al. 2007; Bonnet et al. 2022; Brown et al. 2023). It will be important for future work to extend this characterization to additional Hox genes and to markers specific to presumptive, leg, palp, and antennal tissue identity to better understand the nature of these transformations and to establish conclusively (rather than visually) the molecular identity of these transformed tissues.

### RNAi to *caly* in the differentiating eye causes mild overgrowth

*GMR-gal4* drives expression in cells posterior to the morphogenetic furrow in the differentiating eye (Hay et al. 1994; Freeman 1996) and has also been described to express in the wing, midgut, salivary glands, and trachea (Li et al. 2012; Ray and Lakhotia 2015; Escobedo et al. 2021). Inducing RNAi with *caly^HMC04109^* and *GMR-gal4* did not affect survival (Fig. 4a) and resulted in eyes of apparent normal morphology but increased eye area (quantified in Fig. 4b, eye examples Fig. 4d and F) compared to controls (Fig. 4c and e). Future work should explore if this increase in eye size results from an increase in the number of cells (for example due to increased proliferation or a decrease in apoptosis) or other mechanisms such as dysregulation of organ size homeostasis. Based on the striking differences between eye phenotypes depending on driving RNAi with *ey-gal4* (Fig. 2) or with *GMR-gal4* (Fig. 4), we speculate that Calypso might play different roles in early developing, actively proliferating tissue vs its roles in primarily differentiated tissue.

### RNAi to *caly* in the dorsal wing caused wing vein abnormalities, shriveling, and “cupping”

*ms1096-gal4* drives expression in the dorsal region of the wing disc pouch (Guillén et al. 1995; Rodan et al. 2002) but has also been described to drive expression in halteres, eye discs, and weak expression in ventral regions, including the ventral cuticle (Jonchere and Bennett 2013; Shukla et al. 2014). Inducing RNAi with *caly^HMC04109^* did not statistically significantly affect survival (quantified in Fig. 5a). Given the overlap in expression in eye tissues between *ey-gal4* and *ms1096-gal4*, it is unclear why there were no survival effects due to driving RNAi using *ms1096-gal4* despite the striking lethality seen when driving RNAi using *ey-gal4* (Fig. 2a). We speculate that this could be due to differences in the level or precise pattern of the eye-specific expression from each driver or due to *ey-gal4-*directed expression in other tissues that does not overlap with *ms1096-gal4*. *ms1096 > caly^HMC04109^* wings showed a range of phenotypes (Fig. 5d to d´´, g to g´´, apparent size quantified in Fig. 5b and e) including disruption of wing vein pattern and loss of wing veins, blistering, crumpling/shriveling, a “cupped” wing shape, and a reduction in apparent overall wing size (crumpling and “cupping” interfered with measuring exact wing size) compared to controls (Fig. 5c and f). The wing vein abnormalities resemble those seen upon dysregulating other chromatin regulators and homeotic regulators such as HDAC complex components (Barnes et al. 2018), SWI-SNF chromatin remodeling complexes in the presence of tissue damage (Tian and Smith-Bolton 2021), and trithorax group member *Absent small and homeotic 2* (*ash2*) (Amorós et al. 2002). The shriveling/crumpling phenotype resembled phenotypes seen for knocking down or mutating chromatin and homeotic genes, including Asx (Bischoff et al. 2009), *DISCO Interacting Protein 1* (*DIP1*) (Bondos et al. 2004), *L(3)mbt* (Richter et al. 2011), *Antennapedia* (*Antp*) (Fang et al. 2022), and PcG protein *Pleiohomeotic* (*Pho*) (Harvey et al. 2013), and generally appear similar to what has been characterized as a “PcG syndrome” (de Ayala Alonso et al. 2007). The “cupping” phenotype has also been seen upon reduction in HDAC proteins (Barnes et al. 2018) and *ash2* (Amorós et al. 2002), or deleting Polycomb response elements in Polycomb target genes (Sipos et al. 2007). Curiously, while tissue-wide *caly* knockdown resembled these other PcG phenotypes, including by *Asx* mutant clones in the wing (Bischoff et al. 2009), in some contexts, *caly* mutant clones have been reported to cause wing-to-haltere transformation (Bonnet et al. 2022). This may highlight the context-dependent consequences of modulating *caly*.

### RNAi to *caly* across the developing wing caused wing vein abnormalities and curling at the wing margin

*c765-gal4* drives expression across the wing imaginal disc (Guillen et al. 1995; de Celis et al. 1996; Nellen et al. 1996) and also generally in the developing thorax (Gomez-Skarmeta et al. 1996; Yang et al. 2012), in leg discs (Azpiazu and Morata 2002), and in the brain (Rodan et al. 2002). Inducing RNAi across the developing wing with *caly^HMC04109^* and *c765-gal4* resulted in a trend of reduced survival at 21 °C (quantified in Fig. 6a) and this increased and became statistically significant at 25 °C (quantified in Fig. 6f). The increased severity is consistent with presumed higher gal4-mediated transgene expression known to occur with temperature increases due to the temperature responsive nature of the Gal4/UAS system. The wings of flies that survived showed a number of phenotypic abnormalities including loss of wing vein material, a failure of some wing veins to reach the wing margin, buckling of tissue, and curling at the wing margin which was variable at 21 °C (Fig. 6c and e) and increased at 25 °C (Fig. 6h and J) compared to controls (Fig. 6b, d, g, and i). These wing morphology phenotypes resembled but were weaker than those seen for *ms1096-gal4* and similar to those seen for other chromatin regulators described above. Given well-documented patterns for signaling cascades important in wing development and in specification of specific structures like the wing veins or in overall wing size and shape, it will be important for future studies to establish which cells in the developing wing disc are contributing to these phenotypes to help establish the underlying mechanism.

The lethality of driving *caly* RNAi with drivers whose primary expression pattern is in tissues that themselves are not required for viability (e.g. eyes, wings) raises interesting questions regarding the cause of this lethality. It is possible that lethality resulted from effects on other tissues where these Gal4 drivers also induce expression. Another possibility is that this lethality was nonautonomous. A number of *Drosophila* cancer models describe changes in secreted factors upon overexpressing oncogenes or knocking down tumor suppressors. For example, overexpression of oncogenic Yki causes secretion of ImpL2, Pvf1, and Upd3 (Kwon et al. 2015; Song et al. 2019; Ding et al. 2021). In some cases, dysregulation of secreted factors can affect survival, for example by promoting systemic cachexia, coagulopathy, or renal dysfunction (Figueroa-Clarevega and Bilder 2015; Kwon et al. 2015; Hsi et al. 2023; Xu et al. 2023). Therefore, it is possible (i) that *caly* knockdown in these tissues caused a change in the secretion of specific factors that affect survival or (ii) that *caly* knockdown had other systemic or organ effects. As reviewed earlier, BAP1 is a known tumor suppressor and metastasis suppressor, and we noted tumor-like outgrowths in the eye. Therefore, the lethality of *caly* RNAi in certain tissues could also reflect metastasis of cells to other sites. In fact, BAP1 loss is associated with worse prognosis in UM. Pursuing these possible mechanisms in future work may shed light on the requirement for *caly/BAP1* in development with implications for disease.

In addition to a role in cancer, heterozygous mutations in *BAP1* are implicated in a neurodevelopmental syndrome known as Küry-Isidor syndrome (KURIS). This is characterized by developmental delay that affects walking and speech (Küry et al. 2022). Although we cannot rule out off-target effects resulting from RNAi allele *caly^HMC04109^* in the differentiating eye (Fig. 4) or the wing (Figs. 5 and 6), eyes containing primarily *caly^2^* and *caly^C131S^* (Fig. 3) phenocopied driving *caly^HMC04109^* to induce RNAi in the early eye (Fig. 2, Supplementary Fig. 1) as well as phenotypes associated with loss of other chromatin regulators. This is consistent with the *caly^HMC04109^* allele being a useful tool to assess further the mechanisms underlying the role of BAP1 defects in cancer and in Küry-Isidor syndrome. Importantly, some eye outgrowths upon *caly* loss using both RNAi (Fig. 2, Supplementary Fig. 1) and mutant alleles (Fig. 3) resemble tumor-like growths seen for other cancer models (Brumby and Richardson 2003; Pagliarini and Xu, 2003; Uhlirova et al. 2005; Yan et al. 2009; Ho et al. 2015) while others resemble homeotic transformations. Therefore, recapitulating *caly* loss in the early eye using *caly^HMC04109^*, *caly^2^*, and *caly^C131S^* could make an excellent developmental context to study the relationship between epigenetic dysregulation and tumor initiation to be pursued in future work.

## Supplementary Material

jkaf227_Supplementary_Data

## Data Availability

*Drosophila* strains used in this work (listed in Table 1) have been published previously (de Ayala Alonso et al. 2007; Bonnet et al. 2022) or are available from public stock centers. Raw data, normalized data for graphs in Figs. 2 to 6, and *P* values are listed in Supplementary File 1. The authors affirm that all data necessary for interpreting the data and drawing conclusions are present within the article text, the figures, table, and Supplementary File 1. Supplemental material available at G3 online.
