# Supplementary material for: Knockdown of PR-DUB subunit calypso in the developing Drosophila eye and wing results in mis-patterned tissues with altered size and shape
Source: G3 (Bethesda). 2025 Sep 29;15(12):jkaf227. doi: 10.1093/g3journal/jkaf227 (PMC12693516; doi:10.1093/g3journal/jkaf227)
Supplement: jkaf227_Supplementary_Data [file jkaf227_supplementary_data.zip › Supplementary_Figures_G3-2025-406195.docx]

**SUPPLEMENTARY FIGURES for:**

Knockdown of PR-DUB subunit *calypso* in the developing *Drosophila* eye and wing results in mis-patterned tissues with altered size and shape

**Supplementary Figure 1: Additional images of ey>*caly^HMC04109^* heads highlighting outgrowths**. (A-E) Additional examples of ey>*caly^HMC04109^* heads showcasing outgrowths (solid arrows) and bristle abnormalities. (A-A’) Alternate views of the same head showing outgrowths on both sides. (B) Another example of a head with an outgrowth where the outgrowth lacks obvious morphology and is hard to classify. (C) Example of a case where almost an entire leg is growing out of the head with additional tissue on the tip including ommatidia. We have seen multiple cases where outgrowths resemble leg segments, however, this is the only case we have seen where the outgrowth is this long. (D-D’) Alternate views of another head, with an enlarged view in D'. (E-E’) Alternate views of head shown in Fig. 2F-2F’ (enlarged in E’) highlighting the outgrowths. Females are shown in A-A’, C-E, and a male is shown in B.

**Supplementary Figure 2: Decreased *caly “*flattens*”* heads as shown by increased ratio of width-to-height.** (A) Diagram indicating how head height and width were measured for graphs in Fig. 2I-2J and Fig. 3J-3K. Anterior image of a head with lines overlaid. Vertical line indicates how head height measurements were taken for graphs in Fig. 2I and Fig. 3J and horizontal line indicates how head width measurements were taken for graphs in Fig. 2J and Fig. 3K. (B) Graph indicating the Ratio of width-to-height for ey>*caly^HMC04109^* (pink, second and fourth lanes) versus *ey-gal4/+* (black, first and third lanes) heads for males (lanes one and two) and females (lanes three and four). (C) Graph indicating the Ratio of width-to-height for heads containing largely *FRT42D* control tissues (black bars, first and fifth lanes), *Asx^22P4^* mutant tissues (blue, second and sixth lanes), *caly^2^* mutant tissue (dark red, third and seventh lanes), and *caly^C131S^* mutant tissues (fourth and eighth lanes) for males (lanes one through four) and females (lanes five through eight). Statistical analysis for individual height and width measurements is shown in the main figures and summarized in Supplementary File 1. Error bars for the ratios in B-C reflect additive relative standard error of the mean (SEM) based on SEM for height and width calculations. The SEM for the height and width from data in graphs in Figs. 2 and 3 was calculated in GraphPad Prism and then divided by the corresponding means to give relative SEM (e.g., SEM for height divided by mean height to give relative SEM). Relative SEM for height was added to relative SEM for width and then applied to the ratio of width to height.
